# Efficient Chemotherapy of Rat Glioblastoma Using Doxorubicin-Loaded PLGA Nanoparticles with Different Stabilizers

**DOI:** 10.1371/journal.pone.0019121

**Published:** 2011-05-06

**Authors:** Stefanie Wohlfart, Alexander S. Khalansky, Svetlana Gelperina, Olga Maksimenko, Christian Bernreuther, Markus Glatzel, Jörg Kreuter

**Affiliations:** 1 Institute of Pharmaceutical Technology, Goethe-University, Frankfurt, Germany; 2 Institute of Human Morphology, Moscow, Russia; 3 Nanosystem Ltd., Moscow, Russia; 4 Institute of Neuropathology, University Medical Center Hamburg-Eppendorf, Hamburg, Germany; University of California Los Angeles, and Cedars-Sinai Medical Center, United States of America

## Abstract

**Background:**

Chemotherapy of glioblastoma is largely ineffective as the blood-brain barrier (BBB) prevents entry of most anticancer agents into the brain. For an efficient treatment of glioblastomas it is necessary to deliver anti-cancer drugs across the intact BBB. Poly(lactic-co-glycolic acid) (PLGA) nanoparticles coated with poloxamer 188 hold great promise as drug carriers for brain delivery after their intravenous injection. In the present study the anti-tumour efficacy of the surfactant-coated doxorubicin-loaded PLGA nanoparticles against rat glioblastoma 101/8 was investigated using histological and immunohistochemical methods.

**Methodology:**

The particles were prepared by a high-pressure solvent evaporation technique using 1% polyvinylalcohol (PLGA/PVA) or human serum albumin (PLGA/HSA) as stabilizers. Additionally, lecithin-containing PLGA/HSA particles (Dox-Lecithin-PLGA/HSA) were prepared. For evaluation of the antitumour efficacy the glioblastoma-bearing rats were treated intravenously with the doxorubicin-loaded nanoparticles coated with poloxamer 188 using the following treatment regimen: 3×2.5 mg/kg on day 2, 5 and 8 after tumour implantation; doxorubicin and poloxamer 188 solutions were used as controls. On day 18, the rats were sacrificed and the antitumour effect was determined by measurement of tumour size, necrotic areas, proliferation index, and expression of GFAP and VEGF as well as Isolectin B4, a marker for the vessel density.

**Conclusion:**

The results reveal a considerable anti-tumour effect of the doxorubicin-loaded nanoparticles. The overall best results were observed for Dox-Lecithin-PLGA/HSA. These data demonstrate that the poloxamer 188-coated PLGA nanoparticles enable delivery of doxorubicin across the blood-brain barrier in the therapeutically effective concentrations.

## Introduction

Glioblastoma multiforme is the most common and most aggressive type of primary brain tumours in humans accounting for 20% of all intracranial tumours [1]. Chemotherapy of glioblastoma is largely ineffective as the blood-brain barrier (BBB) prevents entry of most anticancer agents into the brain. Conventional methods for enhancing drug concentrations in the brain, such as disruption of the BBB, intraventricular drug injection or local therapy, are highly invasive and, therefore, are not applicable for long-term treatment regimens. Local drug therapy with drugs incorporated into implants or administered by local injection or implantation, on the other hand, suffers from the limited diffusional area that is accessible for the drug released after implantation [2].

One of the most promising approaches, therefore, is the intravenous injection of specially coated nanoparticles that are able to transport drugs across the BBB [3], [4], [5]. Due to this special coating, the nanoparticles adsorb certain blood plasma apolipoproteins [6] that enable an interaction of the particles with the respective lipoprotein receptors located on the brain blood capillary endothelial cells followed by the internalisation of the particles [7] and drug beyond the BBB. Alternatively, other ligands for which receptors exist on these cells or antibodies against these receptors may be bound covalently to the nanoparticles to enable the passage through the BBB [8].

To date, most experiments concerning the treatment of glioblastoma with nanoparticles were performed in rats using doxorubicin-loaded poly(butyl cyanoacrylate) particles coated with polysorbate 80 (Tween® 80) using the extremely aggressive rat glioblastoma 101/8. This glioblastoma model is responsive to chemotherapy and histologically closely resembles human grade IV glioblastoma exhibiting a similarly diffuse growth pattern, high proliferative activity, and considerable necrotization [4], [9].

Other nanocarrier approaches for the treatment of glioblastoma includes the use of nanoconjugates consisting of the biodegradable, nontoxic β-poly(L-malic acid) with bound antisense oligonucleotides and the monoclonal anti-transferrin receptor antibody OX-26. Lee et al. [10] demonstrated the receptor-mediated uptake of these conjugates into endothelial cells using human glioma cell lines in-vitro. No toxicity of this bioconjugate was observed in-vivo in a hemolysis assay. By confocal microscopy, the uptake into the brain was demonstrated. Fujita et al. [11] later revealed a significantly reduced tumour microvessel density and area combined with an increased animal survival using the same bioconjugate after intracranial administration. After intravenous injection Ding et al. [12] evidenced the efficacy of such bioconjugates by a cell viability assay and fluorescence imaging analysis of the drug distribution and tumour accumulation using the Xenogen 200 Living Image System 2.50, as well as by measuring the tumour volume in mice using H&E stained histological slides.

Recently, Gelperina et al. [13] have shown that PLGA nanoparticles coated with poloxamer 188 (Pluronic® F68) may represent an even more promising alternative to poly(butyl cyanoacrylate) nanoparticles, as, in contrast to the latter, the PLGA nanoparticle surface properties as well as the biodegradation rate may be changed by slight alteration in the chemical composition of the polymer. In addition, this material has for a long time been used in clinical practice in the form of implants and as biodegradable injectable microspheres and has a good safety record in humans. In the above mentioned previous paper [13] the efficacy was investigated using the analysis of the survival of rats by Kaplan-Meier plots. This method needs a very long observation period, requires a larger number of animals, and represents an extreme burden on the rats. The alternative method of histological evaluation of the treatment outcomes enables a faster and less animal-burdening evaluation of the anti-tumour effect of the nanoparticle preparations [9], [14].

Therefore, in the present study the influence of alterations in the composition of the PLGA nanoparticles was investigated by determination of the anti-tumour effects of a number of PLGA-based formulations of doxorubicin in the 101/8 rat glioblastoma model employing histological and immunohistochemical methods with the objective to enable a further optimization of this delivery system.

## Materials and Methods

### Materials

The poly(lactide-co-glycolide) polymer Resomer® RG 502H (PLGA, lactide/glycolide = 50∶50, i.v. 0.16–0.24 dl/g) was obtained from Boehringer Ingelheim, Germany. Doxorubicin HCl was purchased from Yick-Vick Chemicals and Pharmaceuticals (Hong Kong). Poloxamer 188 (Pluronic® F68), poly(vinylalcohol) (PVA, MW 30–70 kDa, 88% hydrolyzed), human serum albumin (HSA, fraction V, purity 96–99%, 65,000 Da) and soybean lecithin were purchased from Sigma (Steinheim, Germany). All other chemicals and solvents were of analytical grade.

### Preparation of doxorubicin-loaded PLGA nanoparticles

A 2.5% solution of doxorubicin in 2 ml of Milli-Q water was poured into a solution of 500 mg of PLGA (or 500 mg PLGA and 35 mg lecithin) in 3 ml of dichloromethane. The mixture was emulsified using a high shear rotor stator mixer (Ultra-Turrax T-25, IKA, Germany). The obtained pre-emulsions were then added to 25 ml of 1% aqueous solution of PVA or 1% HSA in phosphate buffer saline (PBS, pH 7.2), and the mixture was passed through a high-pressure homogenizer (Emulsiflex C-5, Avestin Inc., Canada) at 600 bar. Then the organic solvent was removed using a rotary evaporator. The resulting nanosuspension was filtered through a G2 sintered glass filter and freeze-dried after addition of 5% of mannitol used as a cryoprotector. For coating with poloxamer 188 the lyophilized nanoparticles were resuspended before injection in a 1% aqueous solution of poloxamer 188.

### Characterization of the nanoparticles

#### Particle size measurement

The mean particle size was measured by dynamic light scattering using a Malvern Zetasizer 3000 HS_A_ (Malvern Instruments Ltd., Malvern, UK). The measurement was carried out at a cell temperature of 25°C, a scattering angle of 90°; a He-Ne laser (633 nm) was used. The samples were diluted 1∶50 with Milli-Q water.

#### Evaluation of the drug content and drug encapsulation efficiency

For the assessment of the doxorubicin content the lyophilized doxorubicin-loaded nanoparticles were dissolved in DMSO containing 0.004% HCl. After ultrasonication for 20 min, the insoluble material was separated by centrifugation for 15 min at 16,000 g. The concentration of doxorubicin in the supernatant was measured spectrophotometrically at 480 nm with a HELIOS ZETA Spectrometer (Thermo Scientific, UK). The encapsulation efficiency (percentage of doxorubicin bound to nanoparticles) was calculated as the difference between the initial drug content and the amount of free doxorubicin in the filtrate after separation of the nanoparticles by ultrafiltration (Ultrafree MC centrifugal filter units, 30,000 NMWL, Millipore, USA).

#### Release study

The kinetics of doxorubicin release from the different types of the PLGA nanoparticles was investigated in aqueous milieu. The freeze-dried nanoparticles were resuspended in Milli-Q water, and this suspension was diluted 25-fold with water. Then the diluted suspension was incubated at 37°C under constant stirring at 150 rpm. After predefined time intervals (1, 2, 3, 4, 6, and 24 hours) 3 ml aliquots of this suspension were taken, and the nanoparticles were separated by centrifugation (20,000 g for 30 minutes at ambient temperature). The concentration of doxorubicin in the supernatant was measured by spectrophotometry at λmax = 480 nm.

#### Differential scanning calorimetry

The influence of doxorubicin, lecithin, and stabilizers on the thermal behaviour of the PLGA-based formulations was analyzed by differential scanning calorimetry (DSC). The calorimetric measurements were performed using differential scanning calorimeters TA-4000 equipped with a DSC-30 heating cell and DSC823e (Mettler-Toledo, Switzerland) at a heating rate of 20°C/min under argon. The samples were measured in aluminium pans. The temperature range was −50°C to 200°C. A blank aluminium pan was used as reference. Glass transition temperature (Tg) was measured for the Resomer 502H alone, Resomer 502H in combination with doxorubicin or lecithin as well as for the tertiary mixture Resomer 502H – lecithin – doxorubicin. All transition temperatures were reported as the onset of the transition.

### In vivo experiments

The animal experiments were performed in accordance with the German Tierschutzgesetz and the Allgemeine Verwaltungsvorschrift zur Durchführung des Tierschutzgesetzes and were authorized by the Regierungspräsidium Darmstadt (V54-19 c 20/15-F 116/16).

#### Animals

All experiments were carried out using adult male Wistar rats with a body weight of 200±20 g obtained from Harlan Winkelmann GmbH, Borchen, Germany. The rats were caged in groups of three and acclimatized for one week. They received standard laboratory chow and water *ad libitum*.

#### Intracranial inoculation of rat glioblastoma

The intracranial implantation of the tumour, the rat glioblastoma 101/8, was performed using fresh tumour tissue as described by Steiniger et al. [4]. To induce the tumour, a piece of tumour tissue (about 10^6^ tumour cells) from the frozen stock was introduced into the bottom of the right lateral ventricle of donor animals using a tuberculin syringe (B. Braun, Melsungen, Germany). Animals were deeply anaesthetized by intraperitoneal injection of 100 mg/kg ketamine and 10 mg/kg xylazine. Through a midline sagittal incision, a burr hole of 1.5 mm in diameter was made with a dental drill 2 mm lateral to the sagittal midline and 2 mm posterior to the right coronal suture. Surgical glue (Turbo 2000 Kleber Universal, Boldt Co, Wermelskirchen, Germany) was used to close the scalp incision. The animals were sacrificed by carbon dioxide asphyxiation after development of the pronounced clinical signs of illness (usually day 14 to day 18), and the brains were removed. The tumour tissue was excised and homogenized with a scalpel. The fresh tumour tissue was implanted into the brains of new experimental animals as described above.

#### Treatment regimen

Tumour-bearing animals were randomly divided into five groups (n = 6) to receive the following formulations by intravenous injection in the tail vein: (1) doxorubicin bound to PLGA nanoparticles stabilized by PVA and coated with 1% poloxamer 188 (Dox-PLGA/PVA); (2) doxorubicin bound to PLGA nanoparticles stabilized by HSA and coated with 1% poloxamer 188 (Dox-PLGA/HSA); (3) doxorubicin bound to lecithin containing PLGA/HSA nanoparticles coated with 1% poloxamer 188 (Dox-Lecithin-PLGA/HSA); (4) doxorubicin in 1% aqueous solution of poloxamer 188 (Dox-sol) and (5) 1% aqueous solution of poloxamer 188 (P188). The formulations were injected intravenously in the dose of 3×2.5 mg/kg doxorubicin on days 2, 5 and 8 after tumour implantation. The injection volume of P188 solution was the same as for the Dox-sol. The animals treated with doxorubicin formulations were sacrificed on day 18 post implantation. The animals of the control group were sacrificed if they exhibited pronounced signs of illness or on day 18 the latest. The brains were carefully removed and processed for histological analysis.

#### Preparation of histological tissue slides

The preparation of the histological tissue slides was performed as described by [14]. For the histological and immunohistochemical evaluation the brains were fixed in 3.75% zinc formalin solution (Thermo Shandon, Pittsburgh, USA) for at least 48 hours at room temperature and afterwards embedded in paraffin. A routine haematoxylin and eosin (H&E) staining on 5 µm thick sections [15] was performed. Sections were analyzed at the level where the cross-sectional area contained the largest diameter of the tumour, if applicable. For immunohistochemical analysis, 5 µm thick deparaffinated sections were used. Blocking of endogenous peroxidase activity, antigen retrieval, and counterstaining with alum-haematoxylin was performed by a Ventana BenchMark XT automatic staining device (Ventana, Tucson, Arizona, USA) according to manufacturer's instructions. Primary antibodies used were mouse monoclonal antibodies against GFAP (M0761, 1∶200, Dako, Glostrup, Denmark), rabbit polyclonal antibodies against Ki67 (ab15580, 1∶100, Abcam, Cambridge, United Kingdom), and vascular endothelial growth factor (VEFG, sc-507, 1∶1000, Santa Cruz Biotechnologies, Santa Cruz, California, USA) in blocking buffer (5% goat serum/45% Tris buffered saline pH 7.6 (TBS)/0.1% Triton X-100 in antibody diluent solution, Zytomed, Berlin, Germany). Histofine universal immunoperoxidase polymer, anti-rabbit (Nichirei Biosciences Inc., Tokyo, Japan) was used to detect first antibodies. For staining with Isolectin B4, biotinylated Isolectin B4 (B-1205, 1∶30, Vector Labs, Burlingame, California, USA) was used in 100 µg/ml bovine serum albumin (BSA, Sigma-Aldrich, Deisenhofen, Germany) in blocking buffer without goat serum. StreptABComplex HRP duet solution (K0492, Dako) was applied for detection of the lectin. The peroxidase reaction was detected using diaminobenzidine (DAB, Sigma-Aldrich) as chromogen. As a negative control, alternating sections were incubated without primary antibodies or Isolectin B4.

#### Measurement of tumour size

For quantitative determination of the tumour size in each animal the maximal tumour area was determined in serial H&E stained tissue slides using an Axioskop microscope (Carl Zeiss Microimaging, Göttingen, Germany) and a Neurolucida software-controlled computer system.

#### Analysis of proliferation

To assess the proliferating cells, immunohistochemical staining with antibodies against Ki67 was performed. The Ki67- labelling index expressed as the ratio of positively stained tumour cells of all cells was determined from at least six random high power fields (0.19 mm^2^) in each animal.

#### Determination of necrotic areas

For evaluation of necrosis the H&E-stained slides were investigated using a semi-quantitative scoring system: 0, no necrotic area; 1, solitary necroses; 2, less than 50% of the tumour area was occupied by necroses; 3, more than 50% of the tumour cells per area are in the necrotic state.

#### Analysis of blood vessel density

To determine the vessel density, tissue sections were stained with Isolectin B4, a marker for endothelial cells. The percentage of the area occupied by Isolectin B4-positive vessels was quantified with the Axiovision software (Carl Zeiss Microimaging). The areas with the highest vascular density were selected.

#### Investigation of glial fibrillary acidic protein (GFAP) expression

The extent of GFAP expression was assessed semi-quantitatively: 0, no positive staining; 1, ≤20% GFAP-positive cells; 2, ≥20% and ≤50% GFAP-positive cells; 3, ≥50% of GFAP-positive cells.

#### Study of vascular endothelial growth factor (VEGF) expression

The dimension of VEGF expression was evaluated with the following semi-quantitative scoring system: 0, no staining; 1, mild staining; 2, moderate staining; 3, strong staining.

### Statistical analysis

Results are reported as mean values ± standard deviation (SD). Statistically significant differences were evaluated by the non-parametric Kruskal-Wallis-test with post-hoc analysis. Probabilities of *p*≤0.05 were considered as significant.

## Results

### Physicochemical parameters of the nanoparticulate formulations

Doxorubicin is generally used in the form of hydrochloride, which has relatively good solubility in water but is poorly soluble in organic solvents suitable for PLGA nanoparticle preparation, such as dichloromethane or ethylacetate. For this reason, the most appropriate method for loading doxorubicin hydrochloride in the PLGA nanoparticles is the method of double emulsions (W-O-W). As shown by the preliminary experiments, the best results, in terms of doxorubicin loading, were achieved when dichloromethane was used as organic phase and 1% solution of HSA in PBS as outer aqueous phase. This method allowed ∼90% loading of doxorubicin at the drug-to-polymer ratio of 1∶10 (w/w) (Table 1). In contrast, the experiments using 1% HSA solution in water as the external phase yielded only 30–40% loading (data not shown). This phenomenon may be explained by a visibly lower solubility of doxorubicin in PBS as compared to water, probably due to the conversion of doxorubicin hydrochloride into a less soluble doxorubicin phosphate. In any case, this lower solubility facilitated the drug distribution into the organic phase, thus contributing to the efficacy of the encapsulation. Addition of lecithin did not influence the drug loading; however, the mean size of Dox-Lecithin-PLGA/HSA particles was increased as compared to the particles without lecithin: 468 nm versus 380 nm, respectively (Table 1). The PVA-stabilized nanoparticles used in this study had a mean size of 250 nm; doxorubicin loading was 66%.

**Table 1:** Physicochemical parameters of the doxorubicin-loaded PLGA-nanoparticles.

Parameter	Dox-PLGA/PVA	Dox-PLGA/HSA	Dox-Lecithin-PLGA/HSA
Ratio Dox∶Lecithin∶PLGA [w/w/w]	1∶0∶10	1∶0∶10	1∶0.7∶10
Size [nm]	253±3	380±2	468±19
Polydispersity Index	0.179±0.021	0.153±0.035	0.404±0.158
Surface Charge [mV]			
- Before coating	−2.20	−29.3	−25.7
- After coating with P 188	−1.02	−13.3	−11.2
Loading [%]	66.3	90.4	88.5

**Figure 1:**
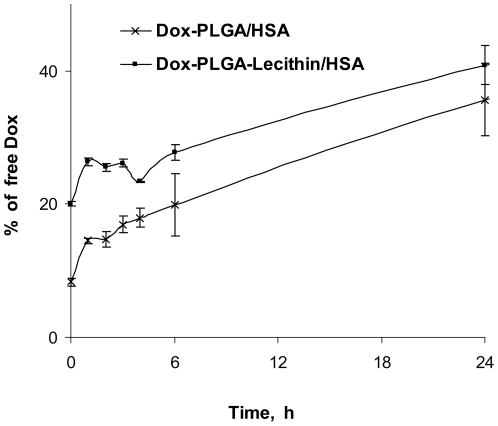
Release of doxorubicin from different types of PLGA nanoparticles stabilized by human serum albumin (water, 37°C, n = 3).

The kinetics of doxorubicin release from the PLGA/HSA nanoparticles displayed biphasic release profiles typical for nanoparticulate formulations (Fig. 1). The initial “burst release” was followed by the second phase, a much slower diffusion-controlled release due to the concentration gradient. These observations correlate with the results of the earlier study [13]. A similar profile was observed for the lecithin-containing particles, although in this case the burst effect was slightly more pronounced. After 60 hours both types of nanoparticles released approximately 60% of doxorubicin.

As shown by the differential scanning calorimetry (DSC) measurements, a slightly faster release of doxorubicin from the Lecithin-PLGA/HSA nanoparticles was not due to the plasticizing effect of lecithin. Table 2 shows the glass transition temperatures (Tg) obtained for the Resomer 502H alone, together with lecithin or doxorubicin as well as the mixture of these components. The DSC thermogram of the polymer alone showed the inflexion point at 56.39°C. The inflexion points of binary mixtures of the polymer with doxorubicin or lecithin exhibited the similarly decreased Tg values of approximately 31°C. Addition of the third component produced only a slight further decrease of the glass transition temperature to 29.03°C. Thus, although the presence of either lecithin or doxorubicin produced a plasticizing effect on the polymer, this effect was not enhanced in the tertiary mixture.

**Table 2:** Glass transition temperatures (Tg) of PLGA (Resomer 502H) and its mixtures with doxorubicin and/or soybean lecithin (differential scanning calorimetry).

Sample composition			
Resomer 502H[mg]	Lecithin[mg]	Doxorubicin[mg]	Tg[°C]
100	-	-	56.39
100	-	10	30.90
100	7	-	31.13
100	7	10	29.03

### Chemotherapy of glioblastoma and histological evaluation

The orthotopic glioblastoma 101/8 has been previously used for evaluation of the anti-tumour efficacy of nanoparticle-bound doxorubicin and appeared to be a brain tumour model that is reliable and sensitive to chemotherapy [16], [17]. Moreover, this tumour model is characterized by a reproducible, invasive growth pattern and displays a close similarity to human glioblastoma [9].

In the earlier study, the considerable anti-tumour effect of the poloxamer 188-coated doxorubicin-loaded PLGA/PVA nanoparticles against this tumour was evidenced by a significant increase of survival time and, even more convincingly, by a number of long-term survivors amounting to 40% (4/10 animals). The HSA-stabilized nanoparticles (Dox-PLGA/HSA) were less effective. The treatment course consisted of three intravenous injections at a dose of 1.5 mg/kg on days 2, 5, and 8 post tumour implantation.

In the present study the anti-tumour effect of the PLGA-based formulations against 101/8 glioblastoma was assessed by histological and immunohistochemical methods using the same tumour model but a more intensive treatment regimen: 3×2.5 mg/kg instead of the previously used 3×1.5 mg/kg. Additionally, Dox-Lecithin-PLGA/HSA nanoparticles were tested. Free doxorubicin in solution (Dox-sol) and 1% poloxamer 188 solution (poloxamer 188 sol) were used as controls. The results of the chemotherapy are presented below.

#### Tumour size

For the evaluation of the tumour size, the maximal tumour area was measured in H&E-stained brain sections of each animal. Table 3 shows the smallest and largest tumour size in each group as well as the mean and standard deviation. In the control group (animals treated with 1% poloxamer 188 solution) the mean tumour area reached 32.1±3.8 mm^2^ (Table 3). The mean tumour size in the group of rats treated with doxorubicin solution was smaller (21.7±13.4 mm^2^); however, the difference between the control and Dox-treated groups was statistically non-significant. The groups treated with Dox-PLGA/PVA and Dox-PLGA/HSA exhibited considerably smaller tumours (10.6±9.7 mm^2^ and 16.6±12.0 mm^2^, respectively). These tumours were distinctively smaller, as compared to controls; however, the difference between these groups and the group treated with Dox-sol was not statistically significant. Significant differences (p≤0.05), as compared to both, control and Dox-sol, were only detectable in the group treated with Dox-Lecithin-PLGA/HSA. The animals in this group exhibited the smallest mean tumour area of 9.6±10.7 mm^2^. Importantly, this was the only group where 2/6 of the animals developed no observable tumour by day 18. The high standard deviation in this group is also caused by this fact. There were no tumour-free animals in other groups.

**Table 3:** Quantitative analysis of tumour incidence, tumour size, proliferation index, and vessel density as well as semiquantitative analysis of GFAP- and VEGF expression after chemotherapy of 101/8 rat glioblastoma with different formulations of doxorubicin.

		Tumour area [mm^2^]						
Group	Incidence of tumour	Mini-mum	Maxi-mum	Mean	Ki 67^+^ [%]	Vessel density [%]	GFAP (score)	VEGF (score)
**Poloxamer 188-sol.**	5/5	28.3	38.8	32.1±3.8	72.2±7.6	3.8±1.0	2.6±0.5	2.4±0.8
**Dox-sol.**	6/6	2.7	34.1	21.7±13.4	63.9±6.6	3.7±1.6	1.7±0.5	1.7±0.7
**Dox-PLGA/PVA-NP**	6/6	0.5	30.3	10.6±9.7	51.2±8.9	2.4±0.5	1.5±0.8	1.7±0.9
**Dox-PLGA/HSA-NP**	5/5	4.0	38.0	16.6±12.0	49.2±8.9	2.3±1.0	2.4±0.5	1.6±0.5
**Dox-Lecithin-PLGA/HSA-NP**	**4/6**	0	27.4	9.6±10.7	34.7±24.8	1.1±1.2	1.0±0.8	0.5±0.5

#### Proliferation index

**Figure 2:**
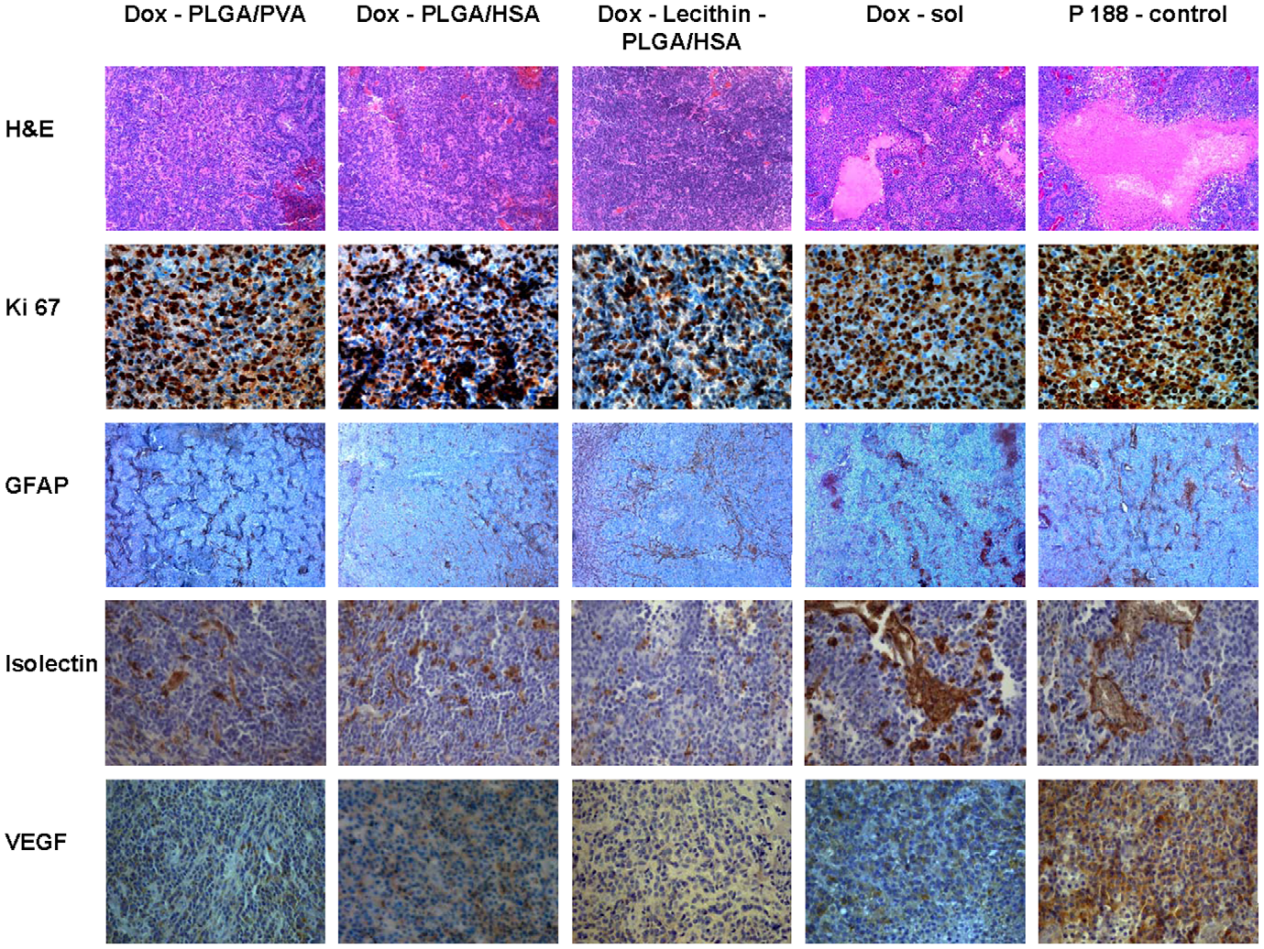
Histological and immunohistochemical evaluation of necrotic areas, proliferation index, GFAP expression, vessel density, and VEGF expression on day 18 after chemotherapy of 101/8 rat glioblastoma with different doxorubicin-PLGA formulations, doxorubicin solution, and poloxamer 188 (P 188) solution as control.

For the evaluation of cell proliferation, immunohistochemical staining with antibodies against Ki67 (MIB-1) was performed. Ki67 is a nuclear antigen, which is expressed in all phases of the cell cycle with the exception of the G_0_-phase. Because of this absence of the antigen in resting cells, it is a good marker for proliferating cells [18]. The Ki67 labelling index expressed as the ratio of positively stained tumour cells of all cells was determined from six random high power fields in each animal. All animals treated with doxorubicin-coated PLGA-nanoparticles showed a significantly lower rate of proliferation than the groups treated with Dox-sol or surfactant solution. The overall best results could be seen in the group treated with Dox-Lecithin-PLGA/HSA where the percentage of proliferating cells amounted to 34.7%±24.8% while the percentage of proliferating cells was 42.7%±21.2% and 49.2%±8.9% in the Dox-PLGA/PVA and Dox-PLGA/HSA treated groups, respectively. The latter difference was, however, statistically not significant. Significantly higher growth rates were detectable in the group treated with Dox-sol where the mean value was 63.9%±6.6%. The highest proliferation rates were found in the control group (72.2%±7.6%) (Table 3). The predominance of positively-stained, brown-coloured cells in the control group and the preponderance of negatively-stained, blue-coloured cells in the successfully treated groups are presented in Fig. 2. Earlier data indicated a significant positive correlation between high Ki67-indexes, high proliferation rates, and a reduction of disease-free intervals as well as shortened survival times [19]. Accordingly, the reduced Ki67-index after treatment with the Dox-Lecithin-PLGA/HSA nanoparticles pointed out impressively the enhanced anti-tumour activity of doxorubicin after incorporation in the PLGA nanoparticles coated with poloxamer 188.

#### Extent of necrotic areas

**Figure 3:**
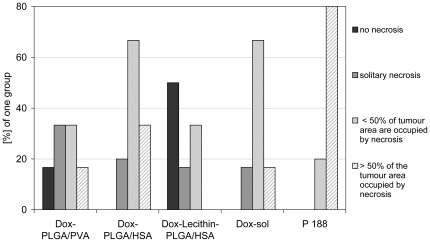
Semi-quantitative analysis of the extent of necrosis after treatment with Dox-PLGA formulations, Dox-sol, and surfactant solution.

An additional diagnostic criterion for high-grade glioblastoma is the presence of large necrotic areas in the brain region occupied by tumour cells. This histological hallmark is a predictor of poor prognosis [20]. The extent of necrosis in different groups is shown in Fig. 2 and 3. Especially widespread necrotic areas with more than 50% of the tumour area occupied by necrosis in 80% of the animals (Score: 2.8±0.4) were found in the group treated with surfactant solution only . An obvious reduction was seen in the groups treated with Dox-sol (2.0±0.6) and Dox-PLGA nanoparticles stabilized with HSA and PVA (2.2±0.7 and 1.5±0.9, respectively). The group of animals treated with Dox-Lecithin-PLGA/HSA was unique in that only solitary necrotic foci could be found mainly in the centre of the tumour (0.8±0.9). In 50% of the rats in this group no necrotic areas were found.

#### GFAP expression

The glial fibrillary acidic protein is a protein of the cytoskeleton and because of its high expression in astroglial tumours, such as astrocytoma and glioblastoma, it is used as a tumour marker [21]. In this study, GFAP expression was evaluated using the following semi-quantitative scoring system: 0, no expression; 1, ≤20% of tumour cells expressing GFAP; 2, between 20% and 50% GFAP expressing cells; 3, ≥50% GFAP expressing cells. The results are summarized in Fig. 2 and Table 3. Histological examination of the control group revealed that each animal showed an expression over 20%, and in 50% of the animals over 50% expression was detectable. Also in the group treated with Dox-sol, in every slide positively stained proteins were found. The average score of the GFAP expression in this group was 1.7±0.5. No significant difference, just a trend, was found between the groups treated with Dox-sol and the particles stabilized with PVA or HSA: regarding the immunoreactive cells for GFAP, the HSA-stabilized particles were superior to the PVA-stabilized particles. The only group exhibiting a significantly reduced GFAP expression was the group treated with the particles containing lecithin. There the GFAP expression did not exceed 50% expression and one third of the rats showed no expression of this marker (1.0±0.8 points).

#### Vessel density

Glioblastomas exhibit high levels of neovascularisation, which contribute to their aggressive behaviour [22]. For determination of the vessel density, tissue sections were stained with Isolectin B4, a marker for endothelial cell. The percentage of the area occupied by Isolectin B4-positive vessels was quantified with the Axiovision software (Carl Zeiss Microimaging). The areas with the highest vascular density were selected. As well as the other histological tests, the analysis of vessel density showed the predominance of the lecithin-containing particles (Table 3). Animals treated with these particles showed a vessel density of only 1.1%±1.1%. In the groups treated with Dox-PLGA/HSA and Dox-PLGA/PVA, the vessel density reached 2.3%±1.0% and 2.4%±0.5%, respectively. A conspicuously higher vessel density was found in the groups treated with Dox-sol and with poloxamer 188 solution: 3.7%±1.6% and 3.8%±1.6%, respectively.

#### VEGF expression

The VEGF is the one of the most important angiogenic factors and a potential prognostic factor for many types of cancer, including glioblastoma [23]. The range of the VEGF expression evaluated using the previously described semi-quantitative scoring system is demonstrated in Table 3. In the control group a strong staining intensity was detectable (2.4±0.8 points). In other doxorubicin-treated groups, the attenuation of expression was evident but not significant. The following scores were found: Dox-PLGA/PVA, 1.7±0.9 points; Dox-PLGA/HSA, 1.6±0.5 points; Dox-sol, 1.7±0.7 points. The lowest VEGF expression was observed in the Dox-Lecithin-PLGA/HSA-treated group (0.5±0.5 points).

## Discussion

As mentioned above, the anticancer antibiotic doxorubicin bound to the PLGA nanoparticles coated with poloxamer 188 produced a considerable anti-tumour effect against intracranial glioblastoma 101/8 in rats [13]. The study also revealed a key role of the surfactant coating as well as the influence of the nanoparticle composition on their biologic performance, i.e. poloxamer 188 appeared to be more effective as a coating agent than polysorbate 80, and the nanoparticles stabilized by PVA appeared to be more effective than those stabilized by HSA.

One of the reasons for this lower efficacy of the HSA-stabilized nanoparticles could be an insufficient attachment of poloxamer 188 to the particles' surface occupied by HSA. It was shown that the poloxamers – block-copolymers of poly(propylene oxide) and poly(ethylene oxide) – interact with cell membranes and, in particular, with lecithin, their essential component [24]. Therefore, it was attempted to improve the efficacy of the HSA-stabilized PLGA nanoparticles loaded with doxorubicin by incorporating lecithin into their core.

Apart from an increase of the particle size, the presence of lecithin did not influence the physicochemical parameters of the HSA-stabilized nanoparticles. However, the considerable advantage of the lecithin-containing particles was clearly seen in the *in vivo* experiment where this formulation demonstrated a superior anti-tumour effect. Although the histological and immunohistochemical results indicated that all PLGA formulations coated with poloxamer 188 were able to transport doxorubicin to the tumour, the Dox-Lecithin-PLGA/HSA produced the most considerable effect on all investigated histological parameters, i.e. the tumour size, proliferation index, GFAP expression, extent of necrotic areas, and vessel density, as well as VEGF expression. The superiority of this formulation compared to the other preparations also is exhibited by the fact that 2/6 animals were tumour-free. This result correlates with the previous finding: considerable inhibition of angiogenesis in 101/8 glioblastoma after chemotherapy using doxorubicin loaded in polysorbate 80-coated poly(butyl cyanoacrylate) nanoparticles [9].

Another outcome of the chemotherapy in this study is the decreased necrotic area. Whereas in general oncology the increase of necrotization indicates an increase of treatment efficacy, the considerable necrotization of human glioma is a sign of its high malignancy and is predictive of a bad prognosis for the patient [20]. Accordingly, the pronounced necrotization of 101/8 glioblastoma is a permanent feature of this tumour. In this case, the decrease of necrotization can be interpreted as a sign of the effective chemotherapy. Indeed, in the present study the absence of necroses or decreased necrotic areas correlated with the decreased tumour sizes. This phenomenon was also observed in the previous study [9].

As mentioned above, chemotherapy also led to considerably decreased vessel density and expression of VEGF. It is known that tumours in the early stages of development (<2 mm) do not yet exhibit an advanced vascular system [25]. Therefore, it is possible that the growth inhibition of small gliomas observed after chemotherapy using doxorubicin-loaded nanoparticles is a result of the inhibited proliferation, which, in turn, causes the delayed formation of hypoxic areas in the tumour core, the major stimulus for angiogenesis and that thus the effect of doxorubicin on vessel density is an indirect rather than a direct one.

It is known that one of the major factors responsible for the biodistribution of intravenously injected colloidal carriers are the plasma proteins adsorbed by these carriers in the blood. In particular, the study of Petri et al. [6] revealed a correlation between the anti-tumour effects of doxorubicin-loaded poly(butyl cyanoacrylate) nanoparticles coated with poloxamer 188 and polysorbate 80 against 101/8 glioblastoma and the high amount of a plasma protein apolipoprotein A-I found on the surface of these particles after incubation in rat plasma. Thus, these two chemically different surfactants obviously yielded a certain similarity of the surface properties of their nanoparticles and mediated similar pharmacological effects, which, remarkably, provided evidence of the effective brain delivery of doxorubicin with these carriers.

On the contrary, in the present study the difference in the antitumour effects between Dox-PLGA/HSA and Dox-Lecithin-PLGA/HSA, both coated with the same surfactant – poloxamer 188, could be due to the diversity in the surface properties of the particles. As shown before by Verrecchia et al. [26], albumin molecules adsorbed to the PLGA nanoparticle surface during the preparation procedure were quickly replaced by other plasma proteins when these particles came in contact with plasma. However, a fraction of HSA remained irreversibly bound to the surface. It can be hypothesized that the higher efficacy of Dox-Lecithin-PLGA/HSA nanoparticles might be due to the surface altered by the presence of lecithin, which, in its turn, could enhance adsorption of apolipoprotein A-I which is known to have affinity to phospholipids [27]. This effect could be enhanced by the aforementioned possible affinity between lecithin and poloxamer 188. The latter proved to be a key ingredient in brain delivery by the PLGA nanoparticles [13].

Together with the data obtained previously, the results of the present study unequivocally demonstrate that PLGA nanoparticles coated with poloxamer 188 enable brain delivery of agents that cannot independently permeate across the blood-brain barrier in therapeutically effective concentrations. In particular, this technology might represent a great potential for non-invasive chemotherapy of brain tumours. The employment of lecithin as an additional ingredient appears to further improve the anti-glioblastoma activity, which may be an indication that further improvements are possible.
